# Mutational landscape changes of AML in patients relapsing after allogeneic hematopoietic cell transplantation

**DOI:** 10.1038/s41409-026-02813-4

**Published:** 2026-04-03

**Authors:** Kristina Maas-Bauer, Thomas Meyer, Mehtap Yücel, Maria Garofalaki, Stefanie Koßmann, Francesca Biavasco, Memnon Lysandrou, Miguel Waterhouse, Tobias Feuchtinger, Dietmar Pfeifer, Florian Ingelfinger, Tobias Wertheimer, Justus Duyster, Jae Sook Ahn, Robert J. Soiffer, Jürgen Finke, Ralph Wäsch, Alexandros Spyridonidis, Dennis Dong Hwan Kim, Claudia Wehr, Robert Zeiser

**Affiliations:** 1 Department of Medicine I, Medical Center - University of Freiburg, Faculty of Medicine, Albert Ludwigs University (ALU), Freiburg, Germany; 2 IMMediate Advanced Clinician Scientist-Program, Department of Medicine II, Medical Center – University of Freiburg and Faculty of Medicine, University of Freiburg, funded by the Bundesministerium für Bildung und Forschung (BMBF, Federal Ministry of Education and Research) - 01EO2103, Freiburg, Germany; 3 Collaborative Research Institute Intelligent Oncology (CRIION), Freiburg, Germany; 4 Department of Internal Medicine, Bone Marrow Transplantation Unit, University Hospital of Patras, Patras, Greece; 5 Department of Pediatrics, Faculty of Medicine, Albert Ludwigs University (ALU), Freiburg, Germany; 6 Department of Hematology-Oncology, Chonnam National University Hwasun Hospital, Chonnam National University, Hwasun-gun, Republic of Korea; 7 Department of Medical Oncology, Dana-Farber Cancer Institute, and Harvard Medical School, Boston, MA USA; 8 Department of Medical Oncology & Hematology, Allogeneic BMT program, Princess Margaret Cancer Centre, University Health Network, and Faculty of Medicine, University of Toronto, Toronto, ON Canada; 9 German Cancer Consortium (DKTK), Freiburg, and German Cancer Research Center (DKFZ), Heidelberg, Germany; 10 Signalling Research Centres BIOSS and CIBSS – Centre for Integrative Biological Signalling Studies, University of Freiburg, Freiburg, Germany

**Keywords:** Cancer therapeutic resistance, Acute myeloid leukaemia, Bone marrow transplantation

## Abstract

Relapse of acute myeloid leukemia (AML) following allogeneic hematopoietic cell transplantation (allo-HCT) remains a life-threatening complication and is influenced by the underlying biology of the AML and possibly by genetic alterations. In this retrospective multicenter study, we evaluated mutational dynamics of AML cells in 57 patients with relapse after allo-HCT. We observed that 68% of patients exhibited genetic instability, characterized by acquisition or loss of mutations, most frequently involving *FLT3-ITD, NRAS*, and *KRAS*, while founding lesions such as *DNMT3A* were usually retained. Clonal evolution patterns varied, with constant profiles (35.0%), linear (29.8%), branching (22.8%), and parallel (12.3%) evolution. However, these evolutionary categories were not associated with differences in progression-free or overall survival. In contrast, relapse timing was highly prognostic: early relapse ( ≤ 6 months) conferred a significant higher mortality risk compared to late relapse, independent of evolution model. Our findings indicate that while relapse after allo-HCT in AML is genetically diverse, timing of recurrence remains the most critical determinant of outcome. Given that certain genetic changes may inform therapeutic options, these findings highlight the relevance of longitudinal molecular monitoring especially during the early post-transplant period.

## Introduction

Acute myeloid leukemia (AML) is a heterogeneous disease characterized by diverse cytogenetic abnormalities and mutations [1–4]. Genetic alterations, such as *FLT3-ITD* and mutations in *ASXL1, BCOR, EZH2, RUNX1, SF3B1, SRSF2, STAG2, U2AF1, ZRSR2*, and *TP53* genes, are associated with an unfavorable prognosis [5]. Mutations in *PTPN11, NRAS, KRAS, NF1, GATA2, TET2, and DNMT3A* genes are also observed, but their role in disease prognosis is controversial. Several of these mutations cause enhanced proliferation and cell survival advantage, while others interfere with cell differentiation, apoptosis, and drug sensitivity [6]. Some mutations, such as *FLT3-ITD* [7], isocitrate dehydrogenase (*NADP(+)) (IDH) 1* [8], *IDH2* [9], enhancer of zeste 2 polycomb repressive complex 2 subunit (*EZH2*) [10] or DOT1-like histone lysine methyltransferase (*DOT1L*) [11] can be targeted using mutation-specific pharmacological interventions. For patients with an intermediate or adverse risk within the ELN risk score, allogeneic hematopoietic cell transplantation (allo-HCT) often remains the only curative option [12]. The allogeneic graft induces the graft-versus-leukemia (GVL) effect, which can control AML cells in a fraction of patients [13]. However, despite the allogeneic immune pressure leukemia cells manage to escape from the immune response in some patients by loss of mismatched HLA molecules [14], upregulation of inhibitory receptors [15], downregulation of HLA class II [16, 17], reduced cytokine production of AML cells induced by FLT3-ITD/ATF4 signaling [18], release of lactic acid [19] and mouse double minute 2 (MDM2) mediated decrease of the p53/TRAIL-R1/2 axis [20]. Recent advances in single-cell DNA-sequencing technology have highlighted the exceptional degree of clonal heterogeneity and complexity in AML. This clonal architecture enables AML to adapt and evolve rapidly under environmental and therapeutic pressures. Prior work has shown that the occurrence of new genetic mutations was not connected to immune escape, while non-genomic alterations in antigen presentation and T cell costimulation are distinct drivers of leukemia immune escape [15]. However, mutational changes may indicate eradication of therapy-sensitive subclones [21]. Consistent with the concept of alloreactive immune pressure, patients with relapse after allo-HCT demonstrate marked alterations in the mutational landscape of the leukemia blasts, compared to AML relapse following chemotherapy alone [22]. These clonal evolution patterns are not static but can shift during disease progression, treatment, and relapse [23, 24]. Linear evolution can give way to branching or parallel evolution, particularly under strong selective pressures, such as chemotherapy or allo-HCT [25]. As a result, relapsed AML may harbor newly dominant clones that either descend from minor subclones present at diagnosis or represent entirely new evolutionary lineages. This dynamic evolutionary landscape complicates disease management but also presents opportunities for more precise, targeted interventions [26].

In this study, we investigated changes in the mutational landscape of patients with AML-relapse following allo-HCT and assessed clinical outcomes related to the pattern of AML evolution and mutational changes.

## Patients and Methods

### Patients and data collection

The present analysis is a multi-center retrospective cohort analysis, including adult patients who were diagnosed with AML and underwent an allo-HCT at the Medical Center of the University of Freiburg, Germany, the Princess Margaret Cancer Center in Toronto, Canada, and the University Hospital of Patras, Greece, between April 2005 and March 2024 and experienced hematologic relapse before December 2024. Only patients with at least one myeloid molecular genetics panel and frozen material available to perform a second myeloid panel were included. For each patient, two myeloid panels were analyzed: one at primary diagnosis or before allo-HCT and one at the time of relapse diagnosis. For analysis of the mutations, an Illumina TrueSight® Myeloid NGS panel (Illumina, San Diego, USA), which allows screening for mutations in 54 genes and hotspots, was utilized for patients from Freiburg (Supplementary Table1). For patients from Toronto, a NGS screening panel with 84 genes and for patients from Patras, an NGS panel screening for at least 38 genes and hotspots was utilized. Intronic or silent variants, as well as known single nucleotide polymorphisms (SNPs) were not included in the analysis. For samples from Medical Center Freiburg University, the limit of detection was, in general, 3% at primary diagnosis and 1% at relapse. For samples from Patras limit of detection was 5%. In total, 57 (26 from Freiburg, 23 from Toronto and 8 from Patras) patients meeting the criteria were enrolled. The characteristics of the patients are described in Table 1. Relapse was defined as hematologic relapse with recurrence of blasts in the bone marrow or blood [27–29]. The analysis was conducted in accordance with the tenets of the Declaration of Helsinki, and all patients gave informed consent for data collection and analysis. Ethics committee, both in Freiburg and Patras, approved the retrospective analysis of patient data (approval number Freiburg: 23-1452-S1, 547/17; approval number Toronto: 16-6337, approval number Patras: 100/27.02.09, 134/ 03.04.09). Laboratory values during the disease course and clinical outcomes were collected from medical records with a data cut-off for patients from Freiburg in June 2025 and for patients from Toronto and Patras on December 31, 2024.

**Table 1:** Patient, disease, and transplant characteristics of patients with hematological AML relapse after allo-HCT.

Variable	Value
**Number of patients**	57
**Median age years**	52,5 (range 18–71 years)
**Sex no. (%)**	
-Female	31 (54%)
-Male	26 (46%)
**AML genetic classification- no. (%) ELN2022**	
-favorable	7 (12.3%)
-intermediate	19 (33.3%)
-adverse	31 (54.4%)
**Initial treatment for AML**	
-cytarabine- based chemotherapy	42 (73.7%)
-HMA	7 (12.3%)
-Upfront	8 (14%)
**Remission prior to allo-HCT (without upfront)**	
-complete remission	37 (75.5%)
-refractory	12 (24.5%)
**Conditioning**	
TCI Score ≤ 2	25 (43.9%)
TCI Score > 2	32 (56.1%)
**Donor type**	
-MRD	20 (35.1%)
-Haploidentical	4 (7%)
-MUD	26 (45.6%)
-MMUD	7 (12.3%)
**GvHD prophylaxis**	
- CSA/MPA ATLG (Grafalon®) 20-30 mg/kg bodyweight	19 (33.3%)
- CSA/MTX	14 (24.6%)
- CSA/Alemtuzumab +/- MPA	6 (10.5%)
- FK506/MTX	7 (12.3%)
- CSA/MPA	2 (3.5%)
- CSA/MPA/PTCy	2 (3.5%)
- Everolimus/MPA/ATG	2 (3.5%)
- Other	5 (8.8%)
**Best response to allo HCT- no. (%)**	
-Complete remission	53 (92.9%)
-Refractory disease	4 (7.1%)
**Pre-emptive treatment with donor lymphocyte infusions**	9 (15.8%)
**Pre-emptive treatment with Sorafenib**	1 (1.8%)
**Median time from allo-HCT to relapse (range)-days**	176 (range 11 -2289)
**Salvage therapy following relapse** All therapies +/- donor lymphocyte infusions	
- HMA/venetoclax	12 (21.1%)
- chemotherapy	21 (36.8%)
- combination therapy	11 (19.3%)
- FLT3 inhibition	2 (3.5%)
- 2^nd^ alloHCT	1 (1.8%)
- 2^nd^ alloHCT with prior salvage therapy	9 (15.8%)
- no therapy	3 (5.3%)
- other	4 (7.0%)
- unknown	3 (5.3%)

*ELN* European Leukemia Network, *MRD* matched related donor, *MUD* matched unrelated donor, *MMUD* mismatched unrelated donor, *CSA* cyclosporine A, *MPA* mycophenolate acid, *ATLG* anti-T lymphocyte globulin, *MTX* methotrexate, *PTCy* post-transplant cyclophosphamide, *HMA* hypomethylating agent

### Endpoints and assessment

For visualization of patient trajectories between diagnosis and relapse, a Sankey diagram was generated. Patients were included if results from both a baseline myeloid NGS panel (at primary diagnosis or prior to allo-HCT) and a relapse panel were available. Patients with “all wildtype” status at baseline were included if a valid relapse panel result was available. Patients were excluded if no valid VAF value was available at diagnosis (*n* = 3) or if no valid VAF value was available at relapse (*n* = 4). After applying these criteria, 50 patients remained for analysis and were visualized in the Sankey diagram. Endpoints of the analysis included overall survival (OS) (time from transplant to death from any cause, censored at last follow-up) and progression-free-survival (time from transplant to relapse, censored at the time of relapse). For some analyses, paired diagnosis-relapse AML samples were classified into four clonal evolution categories based on changes in their mutational profiles [25]: “Linear evolution”—sequential acquisition of mutations over time, typically resulting in replacement of earlier clones by a dominant descendant clone. “Branching evolution”—divergence of a common ancestral clone into multiple genetically distinct subclones, creating a complex, tree-like leukemia architecture with several coexisting lineages**. “**Parallel evolution”—independent acquisition of distinct mutations in different subclones, increasing leukemic genetic diversity. Additionally, we characterized cases with no changes in mutational profile between diagnosis and relapse as “constant”.

### Statistical Analysis

Data were analyzed using GraphPad Software (Version 10.2.2, December 10, 2009) and SAS V9.4 (SAS Institute Inc., Cary, NC, USA) as well as Python 3.11 (Rossum & Drake, 2010) with the packages pandas, matplotlib, scipy, scikit-learn (Pedregosa et al., 2012), and statsmodels. The investigator was not blinded to the group allocation during data analysis. For group comparisons, log-rank tests were used for time-to-event variables. OS rates were estimated and displayed using the Kaplan-Meier method. Two-tailed *p*-value are reported, and a *p*-value < 0.05 was defined as statistically significant.

## Results

### Patient characteristics

Demographic and transplantation-related characteristics are presented in Table 1. The median age was 52.5 years (range 18–71 years), and female patients were slightly predominant (54%). The majority of AML cases had an adverse risk profile (54.4%), 33.3% were intermediate risk, and 12.3% of the patients had a favorable risk profile at initial diagnosis. The vast majority of patients received standard induction chemotherapy containing a cytarabine-based regimen (73.7%), 12.3% patients received hypomethylating agents (HMA) prior to allo-HCT, and 14% patients were transplanted upfront without induction therapy. In line with the adverse risk profile of the AMLs, 24.5% of the patients were refractory to induction therapy. 56.1% received conditioning with a transplant intensity score (TCI) of at least 2, whereas 43.9% of the patients received treatment with an intensity ≤2. Additionally, 45.6% were transplanted with a matched unrelated donor (MUD), 35.1% received a matched related donor (MRD), and 19.3% received a mismatched donor. Graft-versus-host-disease (GVHD)-prophylaxis was performed according to the recommendations of the European Society for Blood and Marrow Transplantation (EBMT) [30]. Most patients (80.7%) received either a combination of cyclosporine A (CSA), mycophenolate acid (MPA) and anti-T lymphocyte globulin (ATLG-Grafalon®) or CSA/methotrexate (MTX) or CSA/Alemtuzumab +/- MPA or FK506/MTX. The best response to allo-HCT was complete remission in 92.9% of the patients, while 7.1% patients had refractory disease. One patient with a known FLT3-ITD mutation received Sorafenib as prophylactic therapy, and nine patients received pre-emptive post-transplant donor lymphocyte infusions, which were given in cases of declining chimerism or rising molecular markers above normal thresholds. Almost all patients received salvage therapy for relapse, most often hypomethylating agents and venetoclax or chemotherapy was used. The median time from allo-HCT to relapse was 176 days (range 11-2289 days). Only a minor fraction of patients exhibited acute (a)GVHD (75.4%) or chronic (c)GVHD (10.5%) (Supplementary Fig. 3A).

### Diverse mutational landscape during relapse

**Fig. 1:**
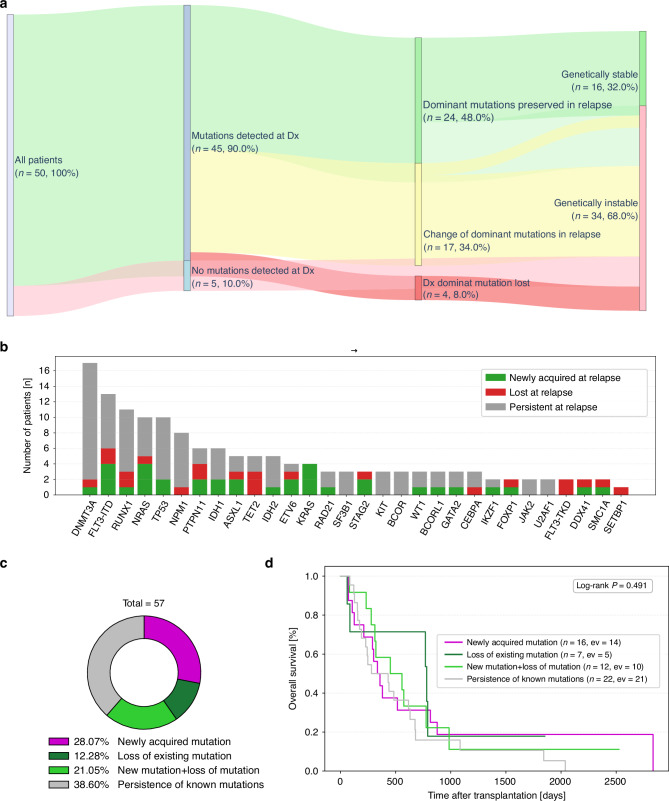
Diverse mutational landscape during relapse. **A** Depicted is a Sankey plot illustrating patient trajectories from diagnosis to relapse (*N* = 50). Patients were first categorized according to mutation status at diagnosis. Among patients with detectable mutations, trajectories were further stratified into loss of the dominant mutation (red), change of the dominant mutation (yellow), or preservation of the dominant mutation (light green). Final nodes represent genetically stable cases (green), defined as no newly acquired or lost mutations, or genetically unstable cases (light red), defined as the occurrence of at least one newly acquired or lost mutation at relapse. Percentages are given relative to the total cohort (100% = all patients included in the Sankey analysis). **B** The bar graph depict the dynamics of gene mutations detected by targeted NGS panel analysis at relapse compared with primary diagnosis. For each gene, mutations are categorized as newly acquired at relapse (green), lost at relapse (red), or persistent across both time points (gray). N indicates the total number of patients harboring a mutation in the respective gene. **C** The donut chart illustrates the mutational landscape at relapse among 57 patients. Categories include newly acquired mutations (purple), loss of previously detected mutations (dark green), coexistence of newly acquired and lost mutations (light green), and persistence of known mutations (gray). **D** Kaplan-Meier estimates for OS probability from transplantation to death of any cause, censored at last follow-up for the indicated groups: acquired mutations (purple), loss of existing mutations (dark green), coexistence of newly acquired and lost mutations (new mutation + loss of mutation; light green) and persistence of known mutations (gray). Significance was calculated using the Log-rank Test. *P* ≤ 0.05 was considered significant.

First, we examined the mutational landscape of our patients and found that the majority of patients had changes in their mutations (genetically unstable, 68%) (Fig. 1A), whereas only in 32% of patients were genetically stable, meaning that all mutations were consistent between primary diagnosis and relapse. Among the “genetically unstable” patients, 11.8% of patients lost their dominant mutation (mutation with the highest variant allele frequency) during relapse, and 34% of patients had a change in their dominant mutation. At primary diagnosis, the most commonly mutated genes in our cohort were *DNMT3A, RUNX1, FLT3-ITD, TP53* and *NPM1* (Supplementary Fig. 1A). At relapse, the mutational landscape remained broadly similar, with *DNMT3A, FLT3-ITD,TP53, RUNX1*, and *NRAS* observed most frequently, with each mutation detected in 9 -16 patients (Supplementary Fig. 1A). Here, we found that mutations in *DNMT3A, FLT3-ITD, RUNX1, TP53, NRAS, ASXL1, ETV6, IDH1, PTPN-11, IDH2, KRAS, BCOR, RAD21, STAG2, WT1, BCORL1, GATA2, IKZF1, FOXP1, DDX41 and SMC1A genes* were acquired at the time of relapse, whereas mutations in *NPM1, TET2, SF3B1, KIT, BCOR, CEBPA, JAK2, U2AF1, FLT-TKD and SETB1* were either persistent or lost during relapse (Fig. 1B). Notably, *FLT3-ITD*, *NRAS* and *KRAS* mutations were most often gained at relapse, each appearing de novo in 4 patients (Fig. 1B), while *DNMT3A, TP53 and NPM1* mutations tended to persist, being retained in the majority (15, 8, 7 patients, Fig. 1B). In contrast, *TET2* mutations were lost in 3 patients (Fig. 1B). Taken together, our analysis revealed that 38.6% of patients showed no change in their mutational landscape upon relapse, whereas 28.07% of patients acquired new mutations and 21.05% of patients both gained new mutations and lost previously detected ones and even a smaller proportion of patients (12.28%) lost one or more of their initial mutations without acquiring any new ones (Fig. 1C). To exclude the possibility that the apparent loss of mutations at relapse was due to a lower leukemic burden rather than true clonal loss, we compared the percentage of blasts in peripheral blood or bone marrow between primary diagnosis and relapse. Across the entire cohort, leukemic burden was significantly lower at relapse (*p* = 0.01, Supplementary Fig. 1B). However, among the seven patients who exhibited only loss of mutations at relapse, blast percentages did not differ significantly from the rest of the patient cohort (*p* = 0.52, Supplementary Fig. 1C), indicating that the observed mutation loss was unlikely attributable to differences in disease burden. In terms of survival in these small patient cohorts, we did not observe any survival differences (*p* = 0.49) between the cohorts (Fig. 1D, Supplementary Fig. 1D).

### Relapse evolution patterns show no prognostic impact in AML

**Fig. 2:**
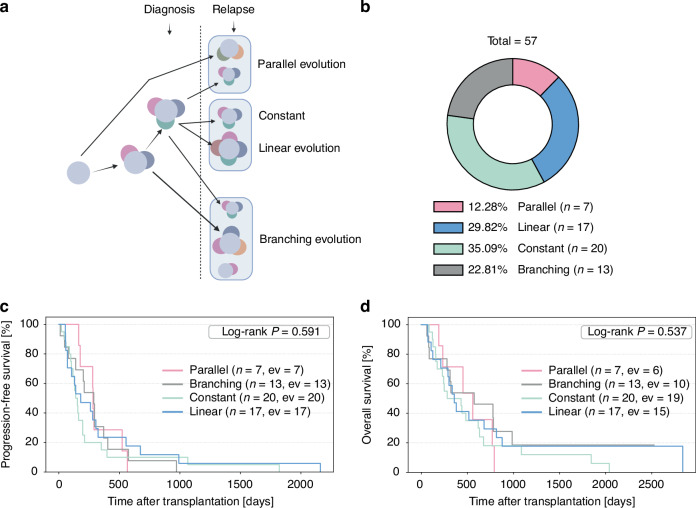
Relapse evolution patterns show no prognostic impact in AML. **A** Depicted are the four clonal evolution categories based on changes in their mutational dynamics during relapse [25]: Parallel evolution: Independent acquisition of distinct mutations in different subclones. Constant: No changes in gene mutations between primary diagnosis and relapse. Linear evolution: Sequential acquisition of mutations over time, typically resulting in replacement of earlier clones by a dominant descendant clone. Branching evolution: Divergence of a common ancestral clone into multiple genetically distinct subclones, creating a complex, tree-like leukemia architecture with several coexisting lineages**. B** The donut chart illustrates the distribution of mutational dynamics at relapse among 57 patients in percent. Evolution patterns include parallel evolution (red), linear evolution (blue), constant gene mutations (light green), and branching evolution (gray). **C** Kaplan-Meier estimates progression free survival from transplantation to relapse, censored at relapse, for the indicated groups: Parallel evolution (red), linear evolution (blue), constant (light green), and branching evolution (gray). Significance was calculated using the Log-rank Test. *P* ≤ 0.05 was considered significant. **D** Kaplan-Meier estimates for OS probability from transplantation to death of any cause, censored at last follow-up for the indicated groups: Parallel evolution (red), linear evolution (blue), constant (light green), and branching evolution (gray). Significance was calculated using the Log-rank Test. *P* ≤ 0.05 was considered significant.

Based on the observation that relapse samples from our patients exhibited diverse mutational changes, we categorized our cohort according to established models of clonal evolution in AML: linear, branching, and parallel evolution [25] (Fig. 2A). The most frequent pattern was a constant mutational composition, observed in 35.0% of cases. Linear evolution was found in 29.8% of cases, while branching evolution was present in 22.8%. Parallel evolution, defined by distinct genotypes in separate subclones, was the least common, occurring in 12.3% of cases (Fig. 2B). To exclude the possibility that group assignment to a specific evolutionary category was influenced by relapse therapy, we analyzed the treatment modalities within the groups and found a heterogenous pattern of treatments in all groups (Supplementary Fig. 1E). We next evaluated whether these clonal evolution patterns were associated with differences in progression-free survival (PFS; Fig. 2C) or overall survival (OS; Fig. 2D, Supplementary Fig. 3D). However, no statistically significant differences in either PFS (*p* = 0.59) or OS (*p* = 0.54) were observed between the groups. We also analyzed whether aGVHD was distributed differently between the specific evolutionary patterns, but did not find any significant differences (Supplementary Fig. 3B).

### Clonal evolutionary patterns and time of relapse

**Fig. 3:**
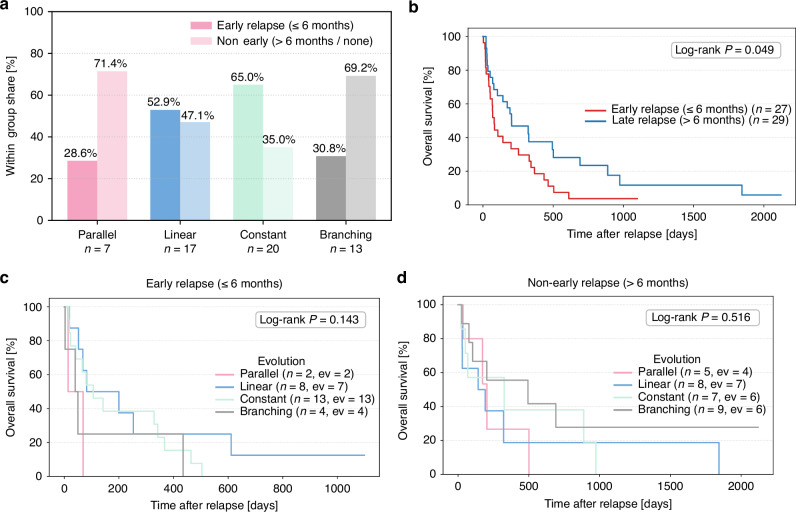
Correlation between clonal evolutionary patterns and timing of relapse. **A** Bar graph showing the proportion of patients experiencing early relapse ( ≤ 6 months, dark bars) versus late relapse ( > 6 months, light bars), stratified by clonal evolution pattern: parallel evolution (red), linear evolution (blue), constant evolution (light green), and branching evolution (gray). **B**. Kaplan-Meier estimates for OS probability from relapse to death of any cause, censored at last follow-up, for patients with early relapse (red) compared to patients with late relapse (blue). Significance was calculated using the Log-rank Test. *P* ≤ 0.05 was considered significant. **C** Kaplan-Meier estimates for OS probability of patients with early relapse ( ≤ 6 months) from relapse to death of any cause, censored at last follow-up for the indicated groups: Parallel evolution (red), linear evolution (blue), constant (light green), and branching evolution (gray). Significance was calculated using the Log-rank Test. *P* ≤ 0.05 was considered significant. **D** Kaplan-Meier estimates for OS probability of patients with non-early relapse ( > 6 months) from relapse to death of any cause, censored at last follow-up for the indicated groups: Parallel evolution (red), linear evolution (blue), constant (light green), and branching evolution (gray). Significance was calculated using the Log-rank Test. *P* ≤ 0.05 was considered significant.

To assess in our patient cohort, whether different evolution patterns are biological factors that result in different intervals to relapse, we stratified patients into those experiencing relapse within the first six months following transplantation (early relapse, n = 28) and those relapsing thereafter (late relapse, n = 29) (Fig. 3). In patients with early relapse, a constant mutational composition was the most frequent evolutionary pattern (46.4%, Supplementary Fig. 2A), with *DNMT3A, FLT3-ITD*, and *NPM1* representing the most common mutations (Supplementary Fig. 2C). In contrast, branching evolution predominated among patients with late relapse (31%, Supplementary Fig. 2B), where *RUNX1, TP53*, and *NRAS* mutations were more frequently observed (Supplementary Fig. 2D).When comparing evolutionary patterns between relapse groups, linear and constant profiles were more prevalent in early relapse, whereas branching and parallel evolution were more common in late relapse (Fig. 3A). To evaluate the prognostic impact of relapse timing, we performed an OS analysis starting at the time of relapse. The 1-year OS of the entire patient cohort following alloHCT was 52.5% Suppementary Fig. 2E) and the 1-year following relapse was 29.7%, whereas the 2-year survival following relapse was 12.7%. (Supplementary Fig. 2F). However, Patients with early relapse had a significantly reduced OS compared to those with late relapse (Fig. 3B; *p* = 0.049). We next analyzed the rate of aGVHD in these two cohorts but did not find any significant differences (Supplementary Fig. 3C). In contrast, OS did not differ significantly between evolutionary patterns, irrespective of relapse timing — either in early relapse (Fig. 3C; *p* = 0.52) or late relapse (Fig. 3D; *p* = 0.73). Collectively, our findings indicate that early relapse after transplantation is associated with markedly inferior survival outcomes.

## Discussion

In this retrospective multicenter study, we assessed the mutational dynamics and clonal evolution patterns of patients with AML who underwent allo-HCT and subsequently developed hematological relapse. Our data revealed that relapse is frequently accompanied by changes in the mutational landscape, but these alterations—and the underlying clonal evolution models they represent–do not translate into measurable differences in post-relapse survival outcomes. It is important to note, however, that the outcome in our cohort was likely influenced by baseline disease severity, as more than half of patients (54%) had ELN adverse-risk AML, and 25% were refractory to therapy before transplantation. These unfavorable baseline characteristics may have contributed to the generally poor survival observed, independent of clonal evolution or mutational patterns at relapse. In contrast, the timing of relapse, particularly within the first six months after allo-HCT, was a strong and independent predictor of poor overall survival, irrespective of specific mutational or evolutionary patterns at relapse.

In line with previous work by Quek and colleagues [31], we observed that the mutational burden had changed at the time of relapse: approximately 61% of patients in our cohort were genetically unstable at relapse, reflecting either acquisition of new mutations, loss of previously detected mutations, or both. This is consistent with prior studies showing that post-transplant relapse can result from the emergence of novel subclones or expansion of pre-existing minor clones that escaped eradication during conditioning and immune surveillance [32]. We observed notable acquisition rates of *FLT3-ITD, NRAS*, and *KRAS* mutations at relapse, highlighting the recurrent activation of proliferative and survival signaling pathways. Conversely, mutations in *DNMT3A and TP53* were frequently retained. These observations are in line with previous studies [33–35] and suggest that certain founding genetic lesions persist throughout the disease course, functioning as clonal “backbones” from which subclonal diversification occurs. The loss of *TET2* and other mutations in a subset of cases is in line with reports that some genetic events, particularly those not critical for leukemic persistence or with growth-suppressive effects in certain contexts, can be lost under post-transplant selective pressures [36].

Classifying relapse events according to established models of clonal evolution [23, 25], we found that constant mutational profiles were most common, followed by linear, branching, and, less frequently, parallel evolution. These evolutionary categories were not tied to specific mutations, specific relapse treatment modalities and did not predict PFS or OS, indicating that diverse genetic pathways can drive disease recurrence after transplant. This finding is in line with our previous work revealing links between certain oncogenic mutations, including *JAK2-V617F*, *KRAS-G12D*, *FLT3-ITD*, *CALR-del52* and distinct immune escape mechanisms, such as PD-L1 expression [37], IL-1β release [38], IL-15 repression [18], galactin-9 expression [39] and TGFβ production [40], respectively. Therefore, future studies, particularly those utilizing single-cell technologies, may be valuable to better characterize and understand the mechanisms underlying disease progression, immune escape and treatment resistance. Of note, the outcomes in all groups may have been influenced by salvage therapies, as most patients in our cohort (51 out of 57) received treatments, such as hypomethylating agents, chemotherapy, Bcl2-inhibition, donor lymphocyte infusion, or participation in clinical trials. In addition, 10 patients underwent a second allo-HCT. Importantly, we found overall very low rates of acute and chronic GVHD across all groups, suggesting limited GVL activity and, consequently, weak immunological pressure—factors that may have facilitated disease relapse.

In contrast, the temporal aspect of relapse carried clear prognostic weight: early relapse within six months of allo-HCT was strongly associated with inferior OS, with a three-fold higher risk of death compared to late relapse. This finding is in line with previous reports [41, 42], and likely reflects the interplay of residual disease burden, immune reconstitution kinetics, and possibly inadequate development of GvL activity in the early post-transplant window. The enrichment of constant and linear evolutionary patterns in early relapse may indicate rapid expansion of pre-existing dominant clones rather than emergence of novel subclones, consistent with the hypothesis that these leukemias never entered deep molecular remission. Conversely, late relapse cases, which more often follow branching or parallel evolution, may arise from more indolently persisting precursor clones that required longer to acquire additional driver lesions or to circumvent sustained GvL mechanisms. Notably, despite these differences in evolutionary trajectories between early and late relapse, we did not observe an enrichment of specific mutations in the early or late relapse group or any significant survival differences among the linear, branching, parallel, and constant evolution groups, which may in part be due to the relatively small number of patients within each subgroup. Due to the retrospective character of our exploratory study, sample sizes remained confined, and heterogeneity with regard to patient characteristics may be present. Yet, our findings emphasize that while post-transplant AML relapse is genetically heterogeneous and shaped by diverse evolutionary trajectories, these patterns alone are insufficient to predict survival without considering relapse timing. From a clinical standpoint, the results are in line with the elegant study from Wienecke and colleagues [33] and highlight the importance of repeated testing using molecular profiling, with particular intensity during the first six months after allo-HCT, when relapse events portend especially poor outcomes. Moreover, our findings illustrate that individual mutations display distinct patterns of acquisition, loss, or persistence at relapse. In particular, mutations that persist at relapse represent especially robust targets for MRD assessment, underscoring the need to account for such evolutionary dynamics when selecting and interpreting MRD monitoring targets.

Future studies with larger patient numbers and longitudinal sampling at multiple post-relapse time points will be needed to more precisely delineate how clonal shifts relate to immune escape mechanisms and microenvironment-based selection. These should be incorporated into registry efforts, such as those conducted by the EBMT, to enable harmonized longitudinal data collection. Integration of multi-omics approaches, including transcriptomic and immunophenotypic profiling, may further unravel why certain clones dominate at specific time intervals after allo-HCT and how this knowledge could be leveraged to improve both preemptive and salvage therapy.

## Supplementary information

Supplemental Table 1.

Revised Supplemental Figure1, Supplemental Figure 2, Supplemental Figure 3, Figure legends (marked up)

## Data Availability

All data are available upon request.
