# Supplementary material for: Mutational landscape changes of AML in patients relapsing after allogeneic hematopoietic cell transplantation
Source: Bone Marrow Transplant. 2026 Apr 3;61(6):677–84. doi: 10.1038/s41409-026-02813-4 (PMC13241310; doi:10.1038/s41409-026-02813-4)
Supplement: Supplementary file 1 — Supplemental Table 1. [file 41409_2026_2813_MOESM1_ESM.docx]

**Supplemental Table 1**

| Patient cohort Freiburg: | | | | | | | |
| --- | --- | --- | --- | --- | --- | --- | --- |
| **Gene** | | | | | | | |
| ABL1 | DNMT3A | KDM6A | RAD2 | CBL | GNAS | NPM1 | STAG2 |
| ASXL1 | ETV6/TEL | KIT | RUNX1 | CBLB | HRAS | NRAS | TET2 |
| ATRX | EZH2 | KRAS | SETBP1 | CBLC | IDH1 | PDGFRA | TP53 |
| BCOR | FBXW7 | MLL | F3B1 | CDKN2A | IDH2 | PHF6 | U2AF1 |
| BCORL1 | FLT3 | MPL | SMC1A | CEBPA | IKZF1 | PTEN | WT1 |
| BRAF | GATA1 | MYD88 | SMC3 | CSF3R | JAK2 | PTPN11 | ZRSR2 |
| CALR | GATA2 | NOTCH1 | SRSF2 | CUX1 | JAK3 |  |  |
| Patient cohort Patras | | | | | | | |
| **Gene** | | | | | | | |
| ASXL1 | CALR | CXCR4 | FBXW7 | IKZF2 | JAK2 | NPM1 | RHOA |
| ATM | CBL | DNMT3A | FLT3 | IKZF3 | MPL | NRAS | RUNX1 |
| BRAF | CEBPA | ETV6 | IDH1 | IKZF1 | MYD88 | PAX5 | SETB1 |
| BTK | CSF3R | EZH2 | IDH2 | JAK1 | NOTCH1 | PCLG2 | SF3B1 |
| SRSF2 | STAG2 | TET2 | TP53 | U2AF1 | ZRSR2 |  |  |
| Patient cohort Toronto | | | | | | | |
| **Gene** | | | | | | | |
| ABL1 | ARID2 | ASXL1 | ASXL2 | ATM | ATRX | BCOR | BCORL1 |
| BRAF | CALR | CBL | CBLB | CBLC | CDKN2A | CEBPA | CREBBP |
| CSF3R | CUX1 | DDX41 | DDX5 | DDX6 | DNAH11 | DNAH5 | DNAI1 |
| DNMT3A | ETV6 | EZH2 | FBXW7 | FLT3 | FOXP1 | GAS2L3 | GATA1 |
| GATA2 | GNAS | HRAS | IDH1 | IDH2 | IKZF1 | JAK1 | JAK2 |
| JAK3 | KDM6A | KT | KMT2A | KMT2C | KRAS | MECOM | MLL |
| MN1 | MPL | MYD88 | NDC80 | NF1 | NOTCH1 | NOTCH3 | NPM1 |
| NR2C2 | NRAS | PDGFRA | PDGFRB | PHF6 | PIGA | PTEN | PTPN11 |
| RAD21 | RB1 | RBM5 | RUNX1 | SETB1 | SF3A1 | SF3B1 | SF3B2 |
| SMC1A | SMC3 | SRSF2 | STAG2 | SUZ12 | TET2 | TP53 | TP63 |
| TP63 | U2AF1 | U2AF2 | WT1 | ZRSR2 |  |  |  |

**Table 1.** Genes evaluated by next-generation sequencing in the different patient cohorts.
