# Supplementary material for: Mutational landscape changes of AML in patients relapsing after allogeneic hematopoietic cell transplantation
Source: Bone Marrow Transplant. 2026 Apr 3;61(6):677–84. doi: 10.1038/s41409-026-02813-4 (PMC13241310; doi:10.1038/s41409-026-02813-4)
Supplement: Supplementary file 2 — Revised Supplemental Figure1, Supplemental Figure 2, Supplemental Figure 3, Figure legends (marked up) [file 41409_2026_2813_MOESM2_ESM.pdf]

Supplemental Figure 1

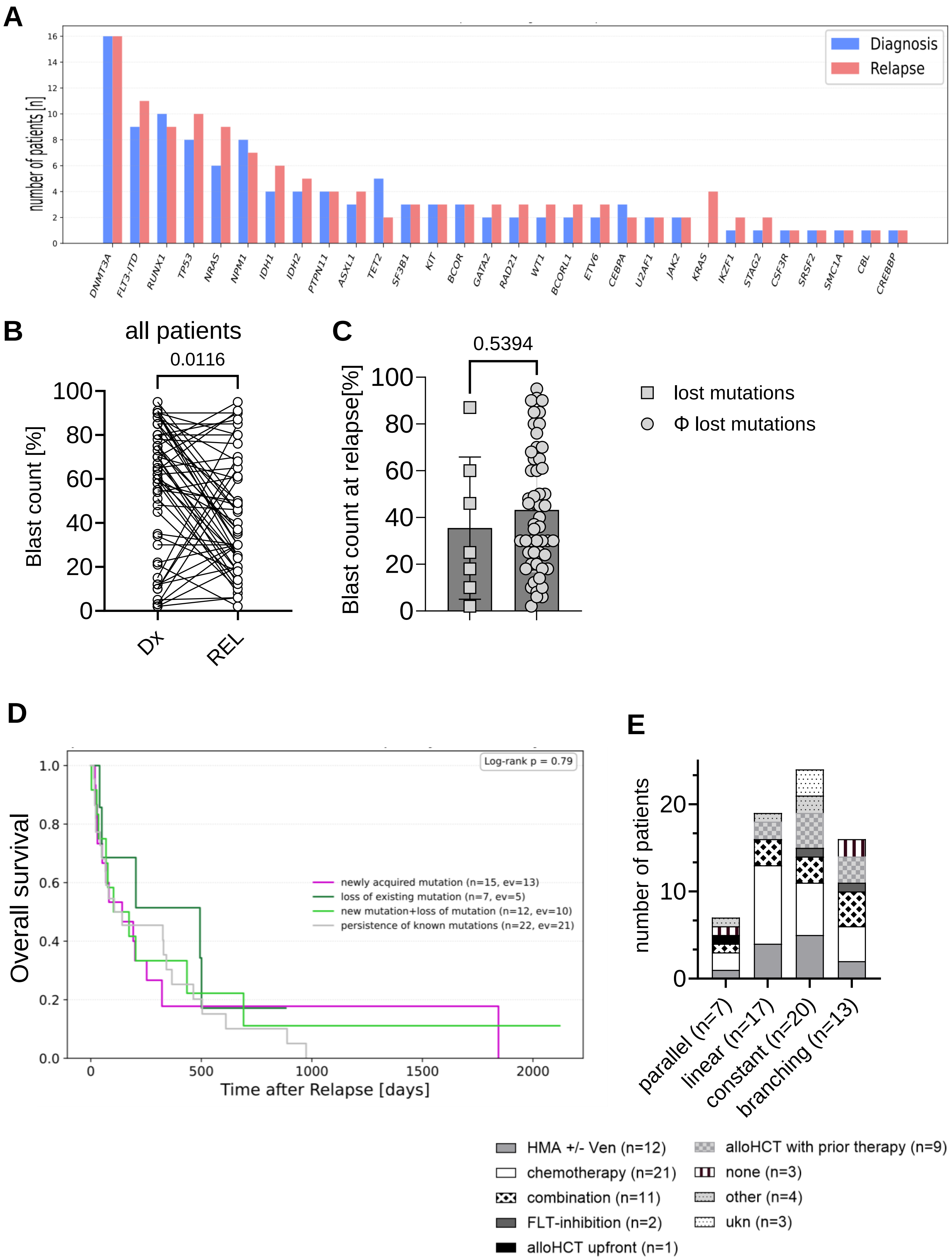

Supplemental Figure 2

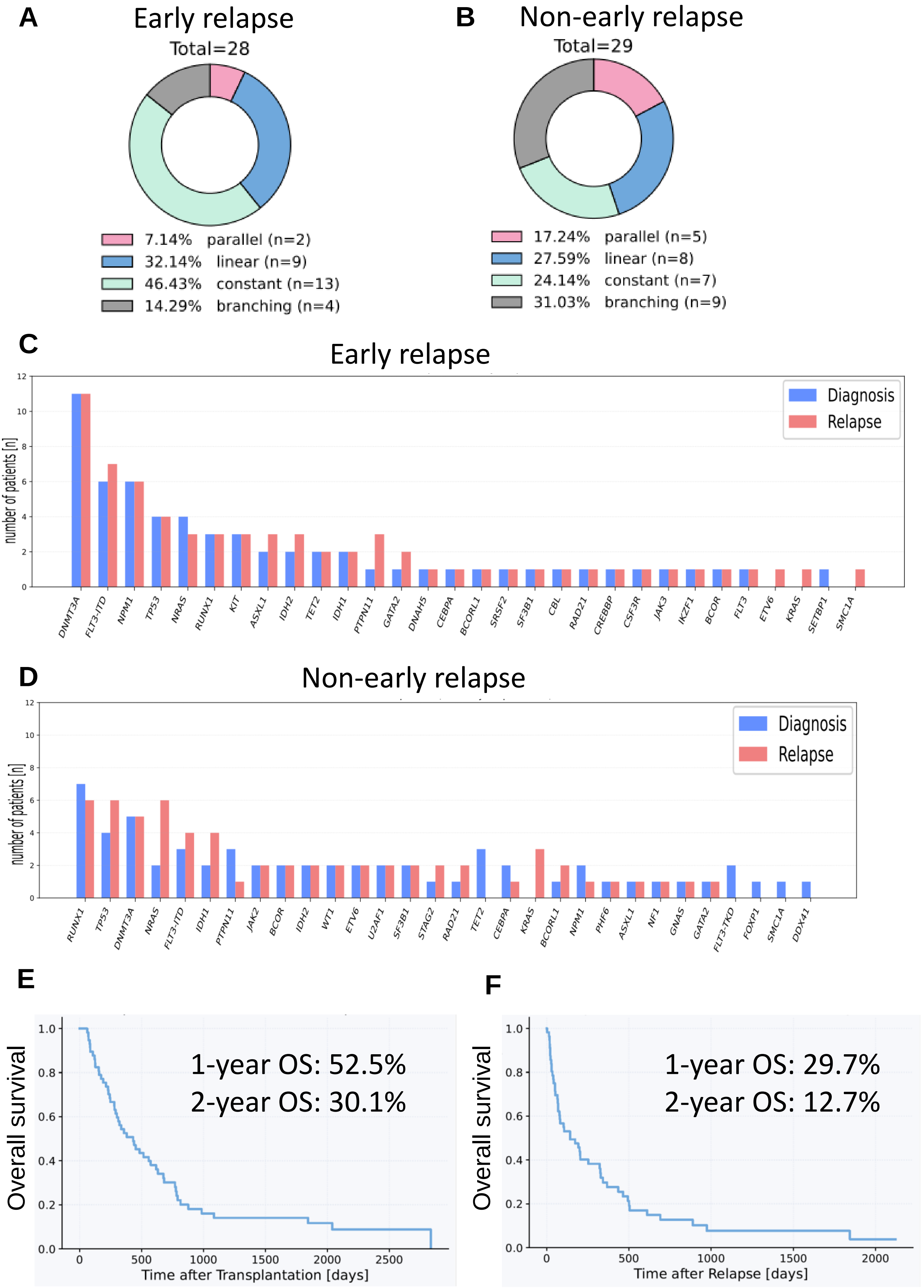

Supplemental Figure 3

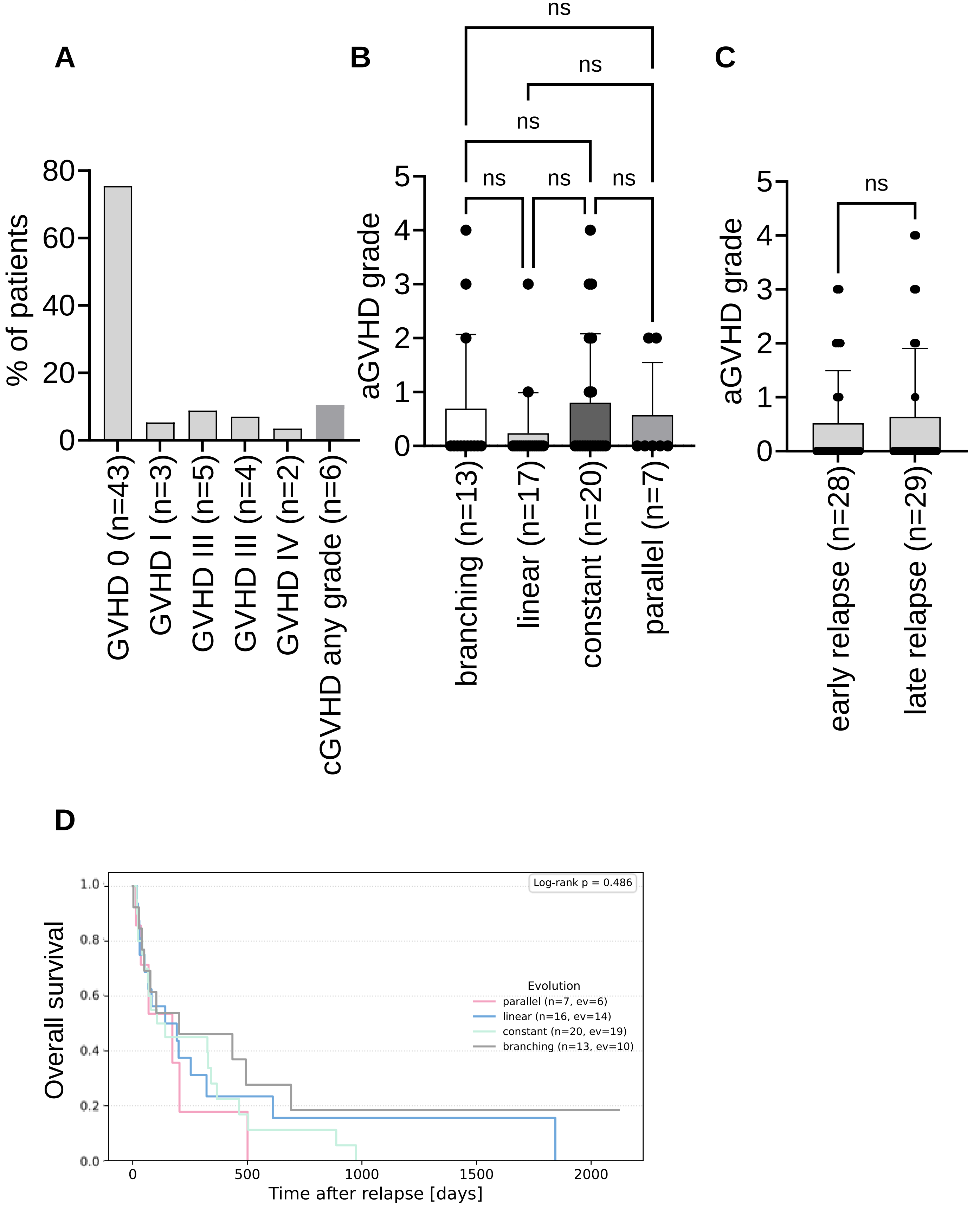

Figure legends

Supplemental Figure 1

A. The bar graph depicts mutated genes detected by targeted NGS panel analysis at primary diagnosis and during relapse. N indicates the total number of patients harboring a mutation in the respective gene.

B. The bar graph shows the blast count [%] at primary diagnosis (Dx) and at relapse (REL). Each dot represents one patient. To calculate significance a paired t-test was performed. $P \leq 0.05$ was considered significant.

C. The bar graph shows the blast count [%] at relapse for patients who lost mutations (square) and patients who did not loose mutations (dot). Each symbol represents one patients. To calculate significance a Welsh's t-test was performed. $P \leq 0.05$ was considered significant.

D. Kaplan-Meier estimates for OS probability from relapse to death of any cause, censored at last follow-up for the indicated groups: acquired mutations (purple), loss of existing mutations (dark green), coexistence of newly acquired and lost mutations (new mutation + loss of mutation; light green) and persistence of known mutations (grey). Significance was calculated using the Log-rank Test. $P \leq 0.05$ was considered significant.

E. The bar graph depicts the total number of patients that were treated with different relapse treatment modalities across the different evolutionary patterns (parallel, linear, constant, branching). N indicates the total number of patients.

Figure legends

Supplemental Figure 2

A. The donut chart illustrates the distribution of evolutional patterns of patients with early relapse (N=28) in percent. Evolution patterns include parallel evolution (red), linear evolution (blue), constant gene mutations (light green), and branching evolution (grey).
B. The donut chart illustrates the distribution of evolutional patterns of patients with non-early relapse (N=29) in percent. Evolution patterns include parallel evolution (red), linear evolution (blue), constant gene mutations (light green), and branching evolution (grey).
C. The bar graph depicts mutated genes of patients with early relapse detected by targeted NGS panel analysis at primary diagnosis and during relapse. N indicates the total number of patients harboring a mutation in the respective gene.
D. The bar graph depicts mutated genes of patients with non-early relapse detected by targeted NGS panel analysis at primary diagnosis and during relapse. N indicates the total number of patients harboring a mutation in the respective gene.
E. Kaplan-Meier estimates for OS probability from transplantation to death of any cause, censored at last follow-up for the entire patient cohort. 1-year OS was 52.5%, 2-year survival 30.1%.
F. Kaplan-Meier estimates for OS probability from relapse to death of any cause, censored at last follow-up for the entire patient cohort. 1-year OS was 29.7%, 2-year survival 12.7%.

Supplemental Figure 3

A. The bar graph depicts the percentage of patients with acute GVHD grade 0,1,2,3 or 4 or chronic GVHD of any grade. N indicates the total number of patients.
B. The bar graph shows the acute GVHD grade of patients in the different evolutionary groups (branching, linear, constant, parallel). Each dot represents one patient and N indicates the total number of patients per group. To calculate significance a 2way ANOVA test was performed. $P \leq 0.05$ was considered significant.
C. The bar graph shows the acute GVHD grade of patients with early relapse (≤6 months) and late relapse (>6months). Each dot represents one patient and N indicates the total number of patients per group. To calculate significance an unpaired t-test was performed. $P \leq 0.05$ was considered significant.
D. Kaplan-Meier estimates for OS probability from relapse to death of any cause, censored at last follow-up for the indicated groups: Parallel evolution (red), linear evolution (blue), constant (light green), and branching evolution (grey). Significance was calculated using the Log-rank Test. $P \leq 0.05$ was considered significant.
